# Supplementary material for: An Automated and Fast Sample Preparation Workflow for Laser Microdissection Guided Ultrasensitive Proteomics
Source: Mol Cell Proteomics. 2024 Mar 20;23(5):100750. doi: 10.1016/j.mcpro.2024.100750 (PMC11067455; doi:10.1016/j.mcpro.2024.100750)
Supplement: Supplemental Figures [file mmc2.docx]

**
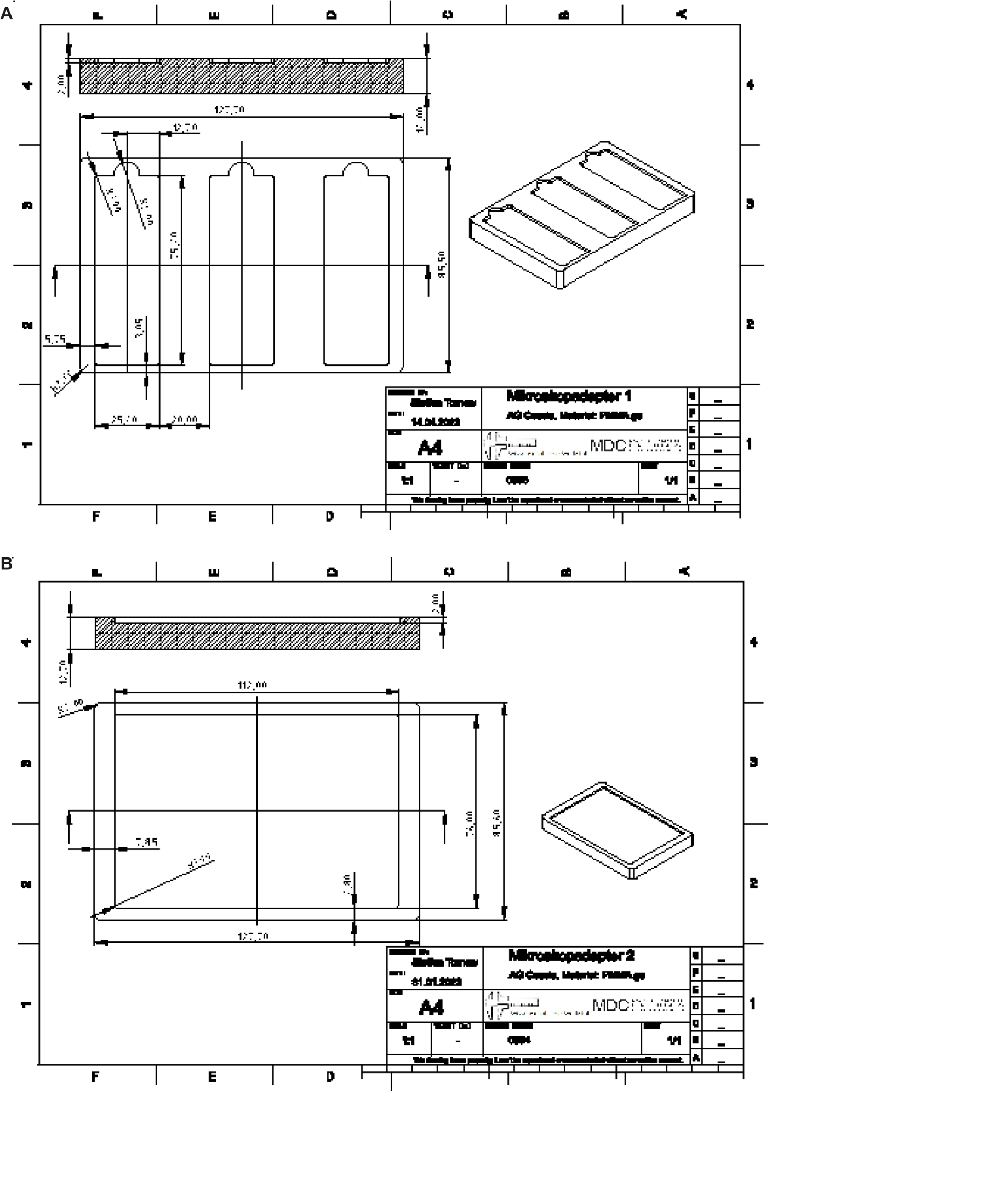
Supplementary Figure 1. Leica LMD7 collection plate adapter design for the cellenONE proteoCHIPs LF 48 and EVO 96.**

**
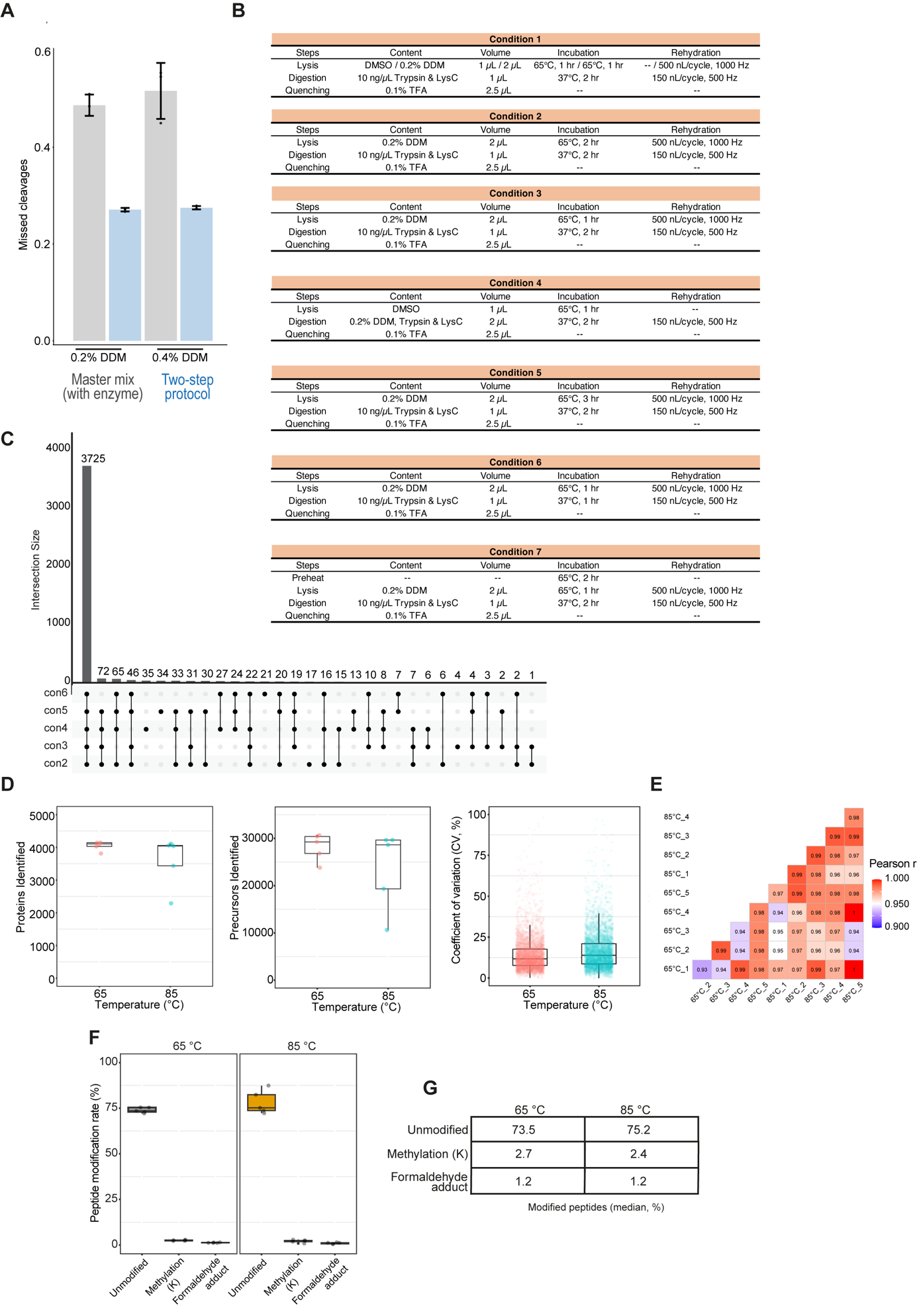
**

**Supplementary Figure 2. Optimizing sample preparation conditions for tissue proteomics on the cellenONE system. (A)** Tryptic miscleavage rates of mouse liver tissue samples processed with four different protocols. Grey bars show the results for the one-step protocol with 0.2% and 0.4% DDM and light blue bars based on a two-step protocol (separated lysis and digestion). Data show mean values from triplicates with standard deviations as error bars. **(B)** Overview of seven different sample preparation protocols tested on the cellenONE system. Laser microdissected mouse liver tissue (50,000 µm^2^) was used as test tissue. **(C)** Upset plot of protein identifications obtained from mouse liver tissue samples processed with the best five protocols (conditions 2-6), depicted in panel (B).

**(D)** Box plots showing the number of identified proteins (left), precursors (center) and the coefficient of variation (CVs) (right) of protein quantification comparing different lysis temperatures. Tissue microregions (50,000 µm^2^) of murine liver were collected for proteomics. CVs were calculated from quadruplicates (65 °C) and quintuplicates (85 °C) of non-log-transformed data. **(E)** Proteome correlation matrix (Pearson’s r) of tissue replicate samples processed using different temperatures. **(F-G)** Open modification search results of murine liver tissues processed using different temperatures during tissue lysis. The boxplot and table show the percentages of unmodified peptides, methylated peptides and peptides with formaldehyde adducts. The box plots (panels D and F) define the range of the data (whiskers), 25th and 75th percentiles (box), and medians (solid line).
